# Atypical Rett syndrome in a girl with mosaic triple X and *MECP2* variant

**DOI:** 10.1002/mgg3.1122

**Published:** 2020-01-13

**Authors:** Satoru Takahashi, Ryo Takeguchi, Mami Kuroda, Ryosuke Tanaka

**Affiliations:** ^1^ Department of Pediatrics Asahikawa Medical University Hokkaido Japan

**Keywords:** *MECP2*, mosaicism, Rett syndrome, X chromosome aneuploidy, X chromosome inactivation

## Abstract

**Background:**

Rett syndrome (RTT) is a neurodevelopmental disorder that predominantly affects girls, resulting from a loss‐of‐function variant in X‐linked *MECP2*. Here, we report a rare case of a girl with RTT with an X chromosome mosaic karyotype (46,XX/47,XXX).

**Methods:**

Fluorescent in situ hybridization (FISH) was carried out to confirm the mosaic karyotype. Sanger sequencing was carried out to genetically diagnose RTT. Furthermore, we assessed the X chromosome inactivation (XCI) pattern. *MECP2* expression levels were examined via RT‐PCR.

**Results:**

The patient presented with preserved speech variant, the milder form of RTT. Genetic examination revealed a de novo, heterozygous, truncating variant of *MECP2*. FISH revealed mosaicism in the 47,XXX karyotype in 6% of her cells. The XCI assay revealed unbalanced inactivation with skewing in favor of the paternal X chromosome. *MECP2* was downregulated to only 84% of the control, indicating that the patient's variant was probably of paternal origin. Unbalanced XCI in this patient might have contributed to the alleviation of the phenotype. However, her supernumerary X chromosome was derived from maternal X chromosome harboring the wild‐type allele and might have had no preferential effect on her RTT‐related phenotype.

**Conclusion:**

The present results indicate that phenotypic effects of X chromosome aneuploidy depend on the nature of the supernumerary X chromosome, the pattern of mosaicism, and XCI status.

## Graphical abstract

We report a rare case of a girl with Rett syndrome with an X chromosome mosaic karyotype (46,XX/47,XXX). The present results indicate that phenotypic effects of X chromosome aneuploidy depend on the nature of the supernumerary X chromosome, the pattern of mosaicism, and XCI status.

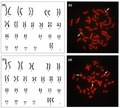

## 1 INTRODUCTION

Rett syndrome (RTT) is a neurodevelopmental disorder predominantly affecting females, clinically manifesting as loss of acquired skills after a period of apparently normal development. RTT results from a loss‐of‐function variant in X‐linked *MECP2* (OMIM #300005), which encodes methyl‐CpG‐binding protein 2 (MeCP2) harboring an N‐terminal methyl‐CpG binding domain (MBD) and a C‐terminal transcription repression domain (TRD).

Females with *MECP2* variants exhibit various clinical signs, ranging from classical RTT to asymptomatic carriers. Psychomotor regression including loss of hand use and communication has been reported in 100% of classical RTT patients (Percy et al., 2010). However, patients with preserved speech variant (PSV), a milder form of RTT, typically recover speech and hand use with time to some extent (Renieri et al., 2009). Most of these affected individuals can speak sentences although autistic features are often observed. Some studies have reported the co‐occurrence of classic RTT and PSV within the same family wherein a girl with classic RTT has a sister affected by PSV (Zappella, 1992). *MECP2* is subjected to X chromosome inactivation (XCI) in females; therefore, the pattern of XCI can influence the phenotypic outcome of variants. Some pairs of phenotypically discordant sisters with classic RTT and PSV have displayed balanced XCI, indicating that other genetic factors beyond XCI potentially contribute to the phenotypic outcome (Grillo et al., 2013).

Males harboring *MECP2* variants have been considered nonviable; however, males with an additional X chromosome (karyotype 47,XXY) potentially present with classic RTT (Leonard et al., 2001). Furthermore, a girl with the 47,XXX karyotype with an *MECP2* variant reportedly presented with mild atypical RTT, suggesting the importance of allele dosage on the severity of RTT phenotype (Hammer, Dorrani, Hartiala, Stein, & Schanen, 2003). Here, we report a rare case of a girl with RTT presenting with an *MECP2* variant and X chromosome mosaic karyotype (46,XX/47,XXX). Her phenotype was mild and consistent with clinical course of PSV. The molecular basis of the milder phenotype of the patient is discussed herein.

## 2 MATERIALS AND METHODS

### 2.1 Patient background and informed consent

The patient was a 12‐year‐old girl with an atypical RTT phenotype, who fulfilled the diagnostic criteria for the disorder (Neul et al., 2010). She and her parents provided informed consent to participate in this study. The experimental protocols were approved by the Committee for Ethical Issues at Asahikawa Medical University.

### 2.2 Karyotype analysis

Peripheral blood samples were obtained from the patient with a heparinized syringe and subjected to karyotyping via G‐banding analysis. Fluorescent in situ hybridization (FISH) was carried out to further confirm the mosaic karyotype using the probe for X alpha‐satellite DNA (DXZ1). In that case, >100 cells were analyzed to evaluate the mosaicism frequency.

### 2.3 Analysis of *MECP2* variants

Genomic DNA was extracted from the peripheral blood leukocytes of the patient and her parents and subjected to PCR analysis. Appropriate primers were used for amplification to yield DNA fragments spanning the entire *MECP2* coding region and intron–exon boundaries, as described in our previous study (Takahashi et al., 2008). The PCR products were analyzed via automated sequencing. For mosaicism analysis, genomic DNA was extracted from various tissue samples of the patient: from buccal mucosa, using a QIAamp DNA Mini Kit (QIAGEN GmbH), and from hair, using an ISOHAIR, (Nippon Gene Co., Ltd.) according to the manufacturer's instructions. To amplify the DNA fragment encompassing the variant site, the following primers were used: for NM_004992.3:c.1157_1197del, forward, 5′‐GTGTCCACCCTCGGTGAGAAG‐3′ and reverse, 5′‐CAGACGCTGCTGCTCAAGTCC‐3′, which generated a 266‐bp product for the wild‐type fragment and a 225‐bp product for the variant fragment. The forward PCR primer was labeled with a fluorescent dye (FAM) to detect the terminal fragment of the PCR products. The PCR fragments were analyzed using ABI 310 automated sequencer, and mutant versus wild‐type peak areas were quantitated using GENESCAN software (Applied Biosystems).

### 2.4 RNA isolation and RT‐PCR analysis

To examine *MECP2* expression levels, total RNA was extracted from peripheral blood cells using the PAXgene Blood RNA Kit (QIAGEN GmbH) in accordance with the manufacturer's instructions. Reverse transcription (RT) was performed using the SuperScript First‐Strand Synthesis System (Invitrogen Corporation) to generate cDNA using 1 μg of total RNA in a 20 μl reaction. Primers used for RT‐PCR were same as those for variant analysis. *GAPDH* was used as an internal control, as described (Takahashi et al., 2008). The PCR products were visualized via ethidium bromide staining, following electrophoresis on 2% agarose gels. The optical densities of the bands were quantified using an image analysis system and ImageJ software (National Institutes of Health; Bethesda, MD).

### 2.5 Analysis of XCI

XCI patterns were determined as previously described (Takahashi et al., 2008). Briefly, aliquots of DNA extracted from the peripheral blood cells were digested overnight using the methylation‐sensitive restriction endonuclease Hpa II. PCR was performed to amplify 100 ng of either digested or undigested DNA using fluorescent PCR primers. The amplified PCR fragment contains an Hpa II site and the highly polymorphic trinucleotide repeat of the androgen receptor gene. As the Hpa II sites on the inactive X chromosome are methylated, only this allele is amplified when the Hpa II‐digested genomic DNA is used as the template. Further, because the methylation site is adjacent to the polymorphic trinucleotide repeat, the alleles amplified by PCR can be distinguished by their length, thus yielding the relative ratio of XCI. The allele peak areas were analyzed using an ABI 310 automated sequencer and GENESCAN software.

## 3 RESULTS

### 3.1 Case report

The patient, a 12‐year‐old girl, was born after 41 weeks without asphyxia following an uneventful pregnancy. Her birth weight and head circumference were 2,880 g (−0.9 *SD*) and 32.0 cm (−1.2 *SD*), respectively. Her psychomotor development was normal during the first 12 months: She acquired head control at 3 months of age, sat without support at 7 months, and spoke meaningful words at 12 months. She could walk alone at 22 months and speak short meaningful sentences at 2 years. Thereafter, her development stagnated, followed by a period of regression. At 3 years of age, she experienced a minor loss in pincer grasp and loss of speech. Some months later, stereotypic hand clapping and hand‐washing activities appeared. From 5 years of age, she gradually recovered some skills of purposeful hand use and speech. She could eat with a spoon and fork; however, she was clumsy and could not press small buttons including those on a television remote control. She walked on a broad base, but could go up and down stairs alone. She could speak a few words in an echolalic manner. At present, she has attended a special school for children with intellectual disabilities. She smiles and maintains eye contact; however, she is passive. No seizures have been reported. At the age of 11 years, her head circumference was 51.8 cm (−1.0 *SD*), her height 141.5 cm (−0.8 *SD*), and her weight 31.1 kg (−1.1 *SD*). No abnormal findings have been observed on brain MRI and on various tests for congenital metabolic disorders.

### 3.2 Chromosomal aberrations

Chromosomal analysis revealed mosaicism with the 46,XX/47,XXX karyotype (Figure S1a,c). FISH analysis confirmed this mosaicism with two different cellular karyotypes: 6% of cells displayed a 47,XXX karyotype, and the remaining 94% displayed a 46,XX karyotype (Figure S1b,d).

### 3.3 Identification of a pathogenic *MECP2* variant

**Figure 1:**
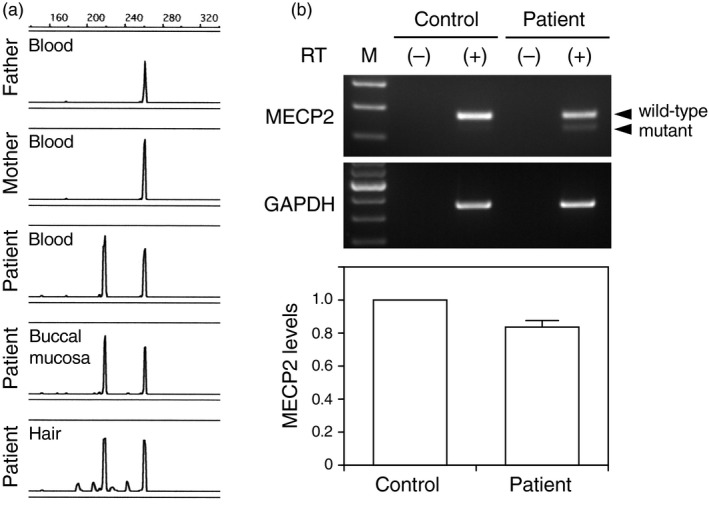
A de novo heterozygous *MECP2* variant and mild downregulation of wild‐type *MECP2*. The PCR product encompassing the variant site shows the wild‐type fragment (266 bp) in the patient's parents' genome and an additional fragment (225 bp) in the patient, which resulted from a 41‐bp deletion (a). Comparable peak heights representing the wild‐type and variant allele in different tissues indicate a heterozygous variant. RT‐PCR analysis reveals a wild‐type fragment (84% of the control levels) with a small amount of the variant fragment in the patient (b). *GAPDH* was used as the internal control. Negative controls without reverse transcription for each PCR reaction were used and yielded no expression

The patient harbored a deletion variant (NM_004992.3:c.1157_1197del) in *MECP2*, which resulted in a shift of the reading frame and introduced a premature stop codon (p.Leu386Hisfs*5). PCR analysis revealed comparable peak heights representing the wild‐type and variant allele in the different tissues, suggesting a heterozygous variant (Figure 1a). Genetic analysis of the patient's parents confirmed that the variant emerged de novo.

### 3.4 *MECP2* expression and XCI analysis

**Figure 2:**
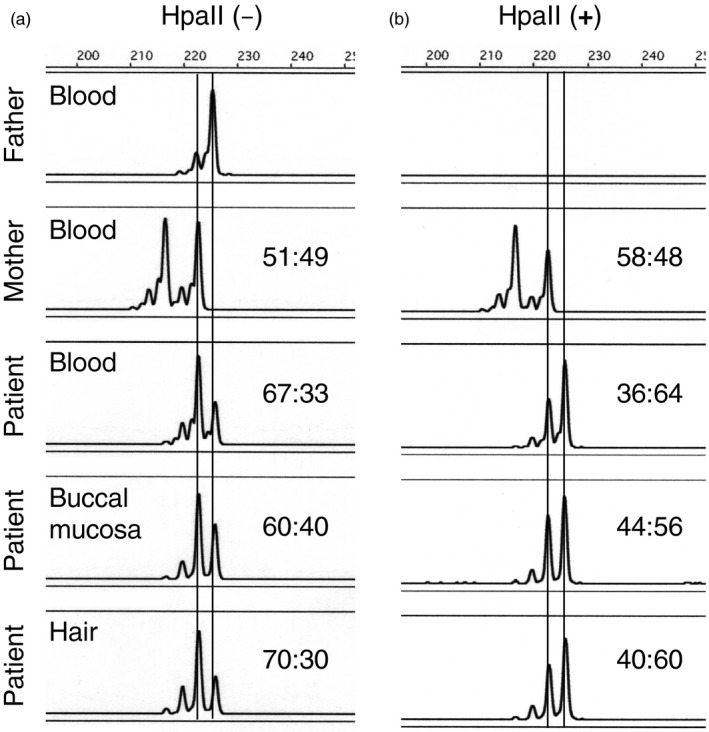
Analysis of X chromosome inactivation (XCI). XCI patterns were determined via the androgen receptor methylation assay. The increased dosage of the maternally derived allele in the undigested samples from various tissues is consistent with the maternal origin of the patient's supernumerary X chromosome (a). Upon digestion with HpaII, the ratio of maternal:paternal alleles inversely decreased owing to skewed inactivation of the paternal X chromosome (b). In each panel, the X‐axis represents molecular masses and the Y‐axis represents the fluorescence intensity of the bands

RT‐PCR analysis revealed that wild‐type *MECP2* was downregulated in the patient to only 84% of the controls, which was greater than expected for a heterozygous variant (Figure 1b). Therefore, we investigated whether a favorably skewed XCI pattern could account for this discrepancy. To assess the XCI pattern of the patient, we assayed the methylation status of androgen receptor alleles (Figure 2). In undigested samples from various tissue specimens including peripheral blood cells, buccal mucosa, and hair, the signal strength ratio of the maternal allele to that of the paternal allele was 60:40 to 70:30, indicating that the supernumerary X chromosome was maternally derived. Upon digestion with HpaII, which permits the amplification of only inactivated alleles, the signal strength of the paternal allele increased relative to the maternal allele: the maternal:paternal allele ratio inversely decreased to 40:60. These results indicated that XCI patterns were unbalanced, favoring expression of the maternal allele, although *MECP2* expression might also be affected by the DNA methylation of the promoter region. The XCI assay and RT‐PCR analysis together reveal mild *MECP2* downregulation, implying that this de novo variant was probably of paternal origin.

## 4 DISCUSSION

This study describes a rare case of a mosaic triple X karyotype in a girl with RTT. The patient presented with PSV, the milder form of RTT. Genetic examination revealed a de novo, heterozygous, truncating variant in the C‐terminal segment of MeCP2, downstream of the TRD. Most PSV patients harbor either missense variants or late truncating variants (Zappella, Meloni, Longo, Hayek, & Renieri, 2001). Early truncating variants tend to result in more severe phenotypes than missense variants, whereas C‐terminal deletions are associated with milder phenotypes (Cuddapah et al., 2014; Smeets, Chenault, Curfs, Schrander‐Stumpel, & Frijns, 2009). The position and type of variant appear to influence the phenotypic outcome. However, even C‐terminal deletions potentially lead to various clinical manifestations, ranging from severe encephalopathy in hemizygous males to classical RTT, PSV, and asymptomatic carriers among heterozygous females (Bebbington et al., 2010; De Bona et al., 2000). Thus, phenotype variability in RTT only partially depends on the type of *MECP2* variant but may rather be attributed to other mechanisms such as skewed XCI and/or modifier gene effects.

We examined the molecular basis of this milder phenotype, particularly focusing on the effect of unbalanced XCI and supernumerary X chromosome. XCI with skewing in favor of the paternal allele (the presumptive mutated allele) might have contributed to the alleviation of the phenotypic outcome. The patient's supernumerary X chromosome was derived from the maternal X chromosome harboring the wild‐type allele, with no preferential effect on her RTT‐related phenotype. Supernumerary X chromosomes overexpress genes that escape XCI; however, in this case of mosaic triple X syndrome, the phenotypic effect might have been limited. However, phenotypes in individuals with X chromosome aneuploidy are affected partly by aberrant expression of escape genes, that is, owing to haploinsufficiency in 45,X cells and overexpression in 47,XXX cells. The phenotypic effects of X chromosome aneuploidy are potentially associated with the occurrence of the mitotic error and the proportion of compromised brain cells.

In conclusion, variable phenotypic severity in RTT with supernumerary X chromosome potentially depends on the nature of supernumerary X chromosome, the pattern of mosaicism, and the XCI status.

## CONFLICT OF INTEREST

The authors declare no conflict of interest.

## Data Availability

The data that support the findings of this study are available from the corresponding author upon reasonable request.
