# Supplementary figures and images for: Atypical Rett syndrome in a girl with mosaic triple X and MECP2 variant
Source: Mol Genet Genomic Med. 2020 Jan 13;8(3):e1122. doi: 10.1002/mgg3.1122 (PMC7057091; doi:10.1002/mgg3.1122)

Figure S1 (Takahashi, et al.)

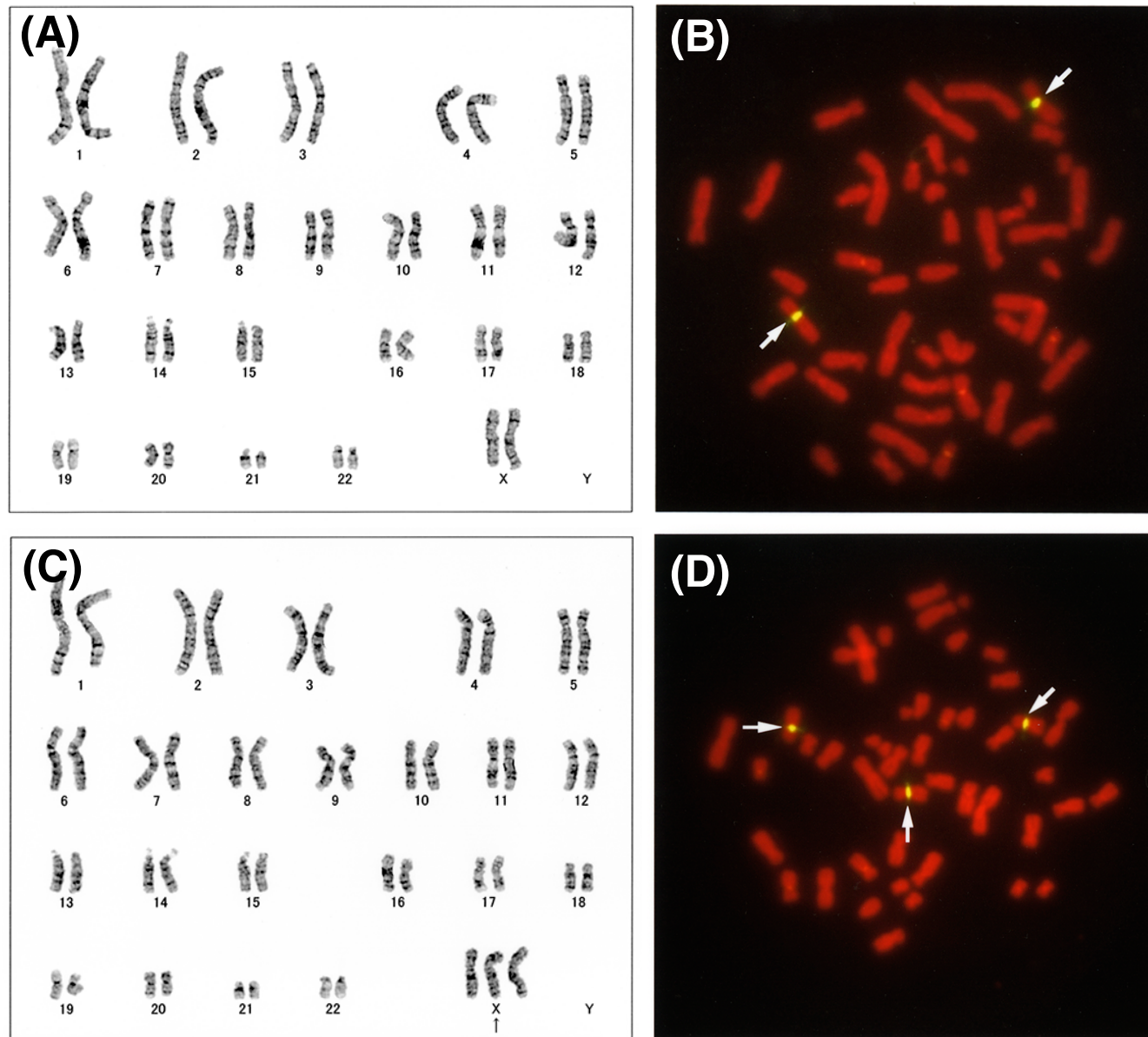

Supplement: Supplementary file 1 [file MGG3-8-e1122-s001.pdf]
